# Circulating and Tissue Biomarkers Associated with Disease Severity and Progression in Adolescent Idiopathic Scoliosis: A Systematic Review

**DOI:** 10.3390/cells15121044

**Published:** 2026-06-06

**Authors:** Francesca Salamanna, Francesca Veronesi, Deyanira Contartese, Giorgia Codispoti, Luca Boriani, Giovanni Tosini, Cristiana Griffoni, Alessandro Gasbarrini, Gianluca Giavaresi

**Affiliations:** 1 Surgical Sciences and Technologies, IRCCS Istituto Ortopedico Rizzoli, 40136 Bologna, Italy; francesca.salamanna@ior.it (F.S.); francesca.veronesi@ior.it (F.V.); giorgia.codispoti@ior.it (G.C.); gianluca.giavaresi@ior.it (G.G.); 2 Department of Spine Surgery, IRCCS Istituto Ortopedico Rizzoli, 40136 Bologna, Italy; luca.boriani@ior.it (L.B.); giovanni.tosini@ior.it (G.T.); cristiana.griffoni@ior.it (C.G.); alessandro.gasbarrini@ior.it (A.G.)

**Keywords:** adolescent idiopathic scoliosis, circulating biomarkers, disease severity, progression, systematic review

## Abstract

**Highlights:**

**What are the main findings?**

- Multiple inflammatory, epigenetic, metabolic, and bone-related biomarkers were consistently associated with AIS severity, particularly with higher Cobb angles and altered skeletal metabolism.
- Only a limited number of longitudinal studies identified potential predictive biomarkers for curve progression, including specific circulating miRNA signatures and reduced spermidine levels.

**What are the implications of the main findings?**

- Biomarkers may improve early risk stratification and support more personalized management of AIS by complementing current clinical and radiographic assessment tools.
- Further large-scale prospective studies with standardized methodologies are required to validate clinically applicable biomarkers for predicting AIS progression and guiding treatment decisions.

**Abstract:**

Adolescent idiopathic scoliosis (AIS) is a multifactorial spinal deformity with variable progression patterns, making early risk stratification challenging. Circulating and tissue biomarkers, including inflammatory, metabolic, endocrine, epigenetic, and bone-related markers, have recently been investigated as potential predictors of disease severity and progression. This systematic review evaluated the current evidence on circulating and tissue biomarkers associated with AIS severity and progression. PubMed, Scopus, and Web of Science were searched for studies published between April 2016 and April 2026. Studies assessing circulating or tissue-based inflammatory, metabolic, epigenetic, and bone-related biomarkers in AIS patients were included. Data on study design, biomarker type, analytical methods, and associations with curve severity or progression were extracted. Twenty-nine studies involving more than 4000 participants were included. Biomarkers identified included inflammatory cytokines, microRNAs, metabolic hormones, and bone metabolism markers. Most studies reported significant associations between biomarkers and curve severity, particularly for inflammatory mediators, epigenetic regulators, and bone-related markers. However, few studies evaluated longitudinal progression, and only a limited number of studies identified predictive biomarkers, including circulating miRNA panels and spermidine levels. ROBINS-I assessment showed substantial risk of bias, mainly related to confounding and selective reporting. Heterogeneity was observed across study designs and outcome definitions. Current evidence supports associations between biomarkers and AIS severity, but predictive value for progression remains limited.

## 1. Introduction

Scoliosis is a three-dimensional deformity of the spine and trunk, defined by a lateral curvature with a major coronal curve measuring ≥10° using the Cobb method on radiographs [1]. Curve severity is commonly stratified into mild (<20°), moderate (20–40°), and severe (>40–50°), with the latter frequently requiring surgical correction [2,3]. In skeletally immature patients, curves exceeding 20° typically prompt closer surveillance and, when indicated, brace treatment to mitigate the risk of progression [3]. Notably, curve progression is highly variable and tends to accelerate during periods of rapid growth, representing a critical window for clinical management [1,3].

Adolescent idiopathic scoliosis (AIS) is the most prevalent form of structural spinal deformity, accounting for approximately 80% of cases [4]. It arises during the pubertal growth phase and may progress until skeletal maturity [1]. Epidemiological data indicate a global prevalence ranging from 1% to 4% among adolescents [1,5,6], with meta-analytic estimates of 1.34% for curves ≥10° [7]. Longitudinal evidence further suggests that up to 2.5% of adolescents develop measurable spinal curvature during growth [8].

Despite its high prevalence, the etiology of AIS remains incompletely understood and is widely considered multifactorial, involving genetic susceptibility alongside environmental, hormonal, metabolic, and biomechanical influences [6,7,8,9]. Disease expression is strongly modulated by sex and growth status, with females exhibiting a markedly higher risk of curve progression and severe deformity. Reported female-to-male ratios range from 1.5:1 to 11:1, depending on curve magnitude and study characteristics [10,11,12].

Current clinical management relies on physical examination and radiographic assessment, including quantification of the major coronal curve using the Cobb method, classification of curve patterns, and assessment of skeletal maturity (e.g., Risser stage or Sanders classification) [13,14]. However, while radiography remains the diagnostic gold standard, it offers limited insight into the biological mechanisms underlying disease progression and provides suboptimal prognostic accuracy [15]. This limitation is clinically relevant, as only a subset of patients will experience significant curve progression. Existing risk stratification approaches, based on age, skeletal maturity, and baseline curve magnitude, lack sufficient precision to reliably identify high-risk individuals at an early stage [16,17,18,19,20,21].

Consequently, treatment decisions are often based on currently available clinical risk estimates rather than individualized biological predictors. Repeated radiographic monitoring also exposes patients to cumulative radiation, while long-term bracing can negatively affect quality of life and psychological well-being [22]. Furthermore, progressive scoliosis may impose a substantial clinical and socioeconomic burden, including aesthetic deformity, chronic pain, functional limitations, reduced quality of life, and pulmonary complications [21,22,23]. Surgical correction, although effective for severe curves, is associated with considerable risks, including neurological, cardiopulmonary, gastrointestinal, and infectious complications, as well as implant-related issues and fusion failure [24,25,26,27].

These limitations highlight the need for objective, minimally invasive tools to improve early risk stratification and disease monitoring. In this context, circulating biomarkers measurable in accessible biological samples have emerged as promising candidates. Among these, inflammatory mediators, including cytokines, interleukins, and other immune-related factors, as well as metabolic and bone-related markers, are increasingly being investigated for their potential role in AIS pathophysiology and progression [28,29,30,31]. These biomarkers may reflect underlying biological processes such as low-grade inflammation, altered bone metabolism, and dysregulated energy balance, which have been associated with scoliosis onset and progression. Although a growing body of literature has explored the relationship between biological biomarkers and AIS, the available evidence remains heterogeneous in terms of study design, patient selection, biomarker assessment, and outcome measures. In particular, the distinction between biomarkers associated with disease severity and those predictive of progression is not always clearly defined [28]. Longitudinal studies provide stronger evidence for predictive value, whereas cross-sectional studies mainly contribute to understanding underlying biological mechanisms.

Therefore, this systematic review aims to comprehensively evaluate the current evidence on circulating and tissue biomarkers associated with disease severity and progression in AIS, highlighting their potential clinical utility and identifying gaps for future research.

## 2. Materials and Methods

### 2.1. PICOS and Eligibility Criteria

This systematic review was designed using the PICOS framework (Population, Intervention, Comparison, Outcomes, Study design) [32]. Specifically, studies were considered if they met the following criteria: P (Population): patients diagnosed with AIS, typically adolescents aged between 10 and 18 years, diagnosed according to standard clinical and radiographic criteria, including Cobb angle measurement; I (Intervention): the exposure of interest consisted of circulating and tissue biomarkers assessed in biological samples from AIS patients. These include circulating biomarkers measured in blood, tissue biomarkers, when available, and non-coding RNAs; C (Comparison): When available, comparisons included differences according to disease severity, curve progression, or non-progressive versus progressive within the AIS population. Comparisons with healthy controls were also considered when reported; O (Outcomes): the primary outcomes were disease severity, assessed through measurements of the major coronal curve using the Cobb method, defined as a significant increase in Cobb angle during follow-up (e.g., ≥5° or ≥10°). Secondary outcomes included the risk of progression, the need for surgical intervention, and associations with growth and skeletal maturity indicators, such as the Risser stage. S (Study design): Eligible studies included observational designs, such as prospective or retrospective cohort studies, case–control studies, and cross-sectional studies in human subjects. Case reports, reviews, editorials, and non-peer-reviewed articles were excluded. Animal and in vitro studies were not considered.

### 2.2. Information Sources and Search Strategy

A systematic search was conducted in March 2026 across three databases (PubMed^®^, Scopus, and Web of Science™), following the Preferred Reporting Items for Systematic Reviews and Meta-Analyses (PRISMA) guidelines [33]. The search strategy is reported in the Supplementary Materials (Table S1).

Filters were used to restrict the results to studies published in English between 2016 and 2026. Duplicate records were removed using EndNote^®^ 2025, and titles and abstracts were independently assessed by four authors (FV, FS, LB, and GT). Studies that did not satisfy the inclusion criteria were excluded. Any discrepancies were resolved through discussion or, when necessary, by consulting a fifth reviewer (DC).

Full-text versions of the remaining studies were then evaluated for eligibility, and their reference lists were also checked for additional relevant publications. Data extraction was carried out independently by FV and FS using a standardized form. The protocol was registered in PROSPERO (Registration number: CRD420261389875).

### 2.3. Risk of Bias Assessment

The risk of bias of the included studies was evaluated using the ROBINS-I (Risk Of Bias In Non-randomized Studies of Interventions) tool [34]. This instrument assesses potential bias across seven domains covering different stages of the study process: bias due to confounding and selection of participants (pre-intervention), classification of interventions (at intervention), deviations from intended interventions and missing data (post-intervention), as well as measurement of outcomes and selection of the reported results. Each domain was judged as having low, moderate, or serious (high) risk of bias, according to the ROBINS-I guidance. Two independent reviewers (FV and FS) performed the assessment, and any discrepancies were resolved through discussion or consultation with a third reviewer (DC) when necessary. The overall risk of bias for each study was determined by the highest level of bias identified in any domain.

## 3. Results

### 3.1. Study Selection

**Figure 1:**
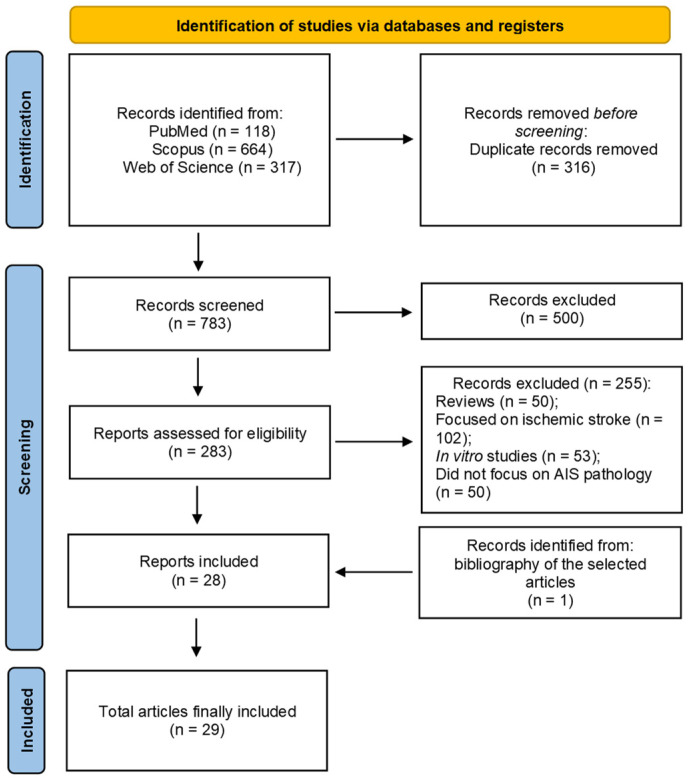
PRISMA flowchart. Search strategy on PubMed, Web of Science and Scopus databases.

The initial database search identified a total of 1099 articles, 118 from PubMed, 317 from Web of Science, and 664 from Scopus. After removing 316 duplicates, 783 studies were screened by title and abstract (Figure 1). Of these, 500 were excluded for not being inherent to the topic. The remaining 283 were assessed for their eligibility and 255 were further excluded for various reasons: 50 were review articles, 102 regarded ischemic stroke, 53 were in vitro studies, and 50 did not focus on AIS pathology. Ultimately, 28 studies met the inclusion criteria, and 1 additional study was identified from reference screening, resulting in a total of 29 studies included in this systematic review [35,36,37,38,39,40,41,42,43,44,45,46,47,48,49,50,51,52,53,54,55,56,57,58,59,60,61,62,63] (Figure 1).

### 3.2. Data Synthesis

The included studies involved over 4000 participants, including both patients with AIS and health controls (Table 1 and Table 2). Study designs were heterogeneous and included cross-sectional [35,36,37,38,39,40,41], case–control [42,43,44,45,46,47,48,49,50,51,52,53,54], prospective [55,56,57], and translational [58,59] approaches. Sample sizes ranged from small exploratory cohorts to large multicenter populations, with most studies showing a predominance of female participants, consistent with the epidemiology of AIS.

A broad spectrum of biomarkers was analyzed across the studies, including inflammatory mediators, epigenetic regulators, metabolic and hormonal factors, and bone-related markers, assessed in serum, plasma, and tissue samples using a variety of analytical techniques (Table 3). Most studies investigated associations between biomarkers and disease severity, typically quantified by the Cobb angle, whereas only a limited number of studies evaluated longitudinal outcomes related to disease progression (Table 4 and Table 5).

Overall, the available evidence is largely derived from cross-sectional analyses, and only a small subset of studies incorporated follow-up data to assess progression. Correlation analyses further highlighted complex interactions between biomarkers and clinical, anthropometric, and metabolic parameters (Table 6). However, substantial heterogeneity in study design, biomarker selection, and outcome definitions was observed across the included studies, limiting direct comparability of findings and the identification of clinically validated biomarkers.

#### 3.2.1. Inflammatory Biomarkers

Several studies investigated the role of inflammatory mediators in AIS, highlighting their association with disease severity. Increased expression of pro-inflammatory cytokines and matrix-degrading enzymes, including interleukin-1β (IL-1β), matrix metalloproteinase-3 (MMP-3), and MMP-13, was observed in association with higher Cobb angles, particularly in severe curves exceeding 70° [35]. Similarly, systemic inflammatory indices derived from routine blood parameters, such as the neutrophil-to-lymphocyte ratio (NLR) and the C-reactive protein/albumin ratio (CAR), were positively correlated with curve severity, indicating a potential role of low-grade systemic inflammation in more advanced deformities [60].

At the molecular level, inflammatory signaling pathways were also implicated. Activation of Toll-like receptor (TLRs) pathways and increased expression of cytokines, including IL-1, IL-6, IL-8, and Tumor Necrosis Factor alpha (TNF-α), were associated with enhanced osteoclastogenesis and tissue degeneration [61]. In addition, immune-related biomarkers such as CD23 and β2-microglobulin were reduced in AIS and showed associations with disease severity [49].

Postoperative increases in inflammatory markers, including IL-6 and C-reactive protein, were observed but were related to surgical stress rather than disease severity or progression [56].

#### 3.2.2. Epigenetic Biomarkers

Epigenetic regulators represented a major class of biomarkers in AIS. Multiple studies identified altered expression of circulating and tissue-specific microRNAs (miRNAs) associated with disease severity. Increased levels of miR-96-5p were associated with AIS and contributed to predictive models including clinical variables [43]. Similarly, elevated expression of miR-941, miR-151a-3p, and miR-148b-5p was observed in more severe cases [51].

Additional studies identified broader miRNA signatures associated with AIS diagnosis and severity. A four-miRNA panel demonstrated high diagnostic accuracy in distinguishing AIS patients from controls [45], while other studies identified multiple miRNA panels associated with severe phenotypes [37,55].

Epigenetic regulation at the DNA and chromatin level was also implicated. Increased estrogen receptor 1 (ESR1) methylation was positively associated with curve severity [36], while histone modifications involving SUV39H1 and H3K9me3 were linked to increased chondrocyte proliferation and AIS severity [46]. Furthermore, altered expression of regulatory genes involved in inflammatory signaling was reported, including reduced SOCS3 expression and genetic variants associated with increased curve severity [62].

Finally, circulating microRNAs such as miR-130b-3p were associated with reduced bone mass and increased disease severity, supporting a link between epigenetic regulation and skeletal metabolism [41].

In addition, extracellular vesicle-associated microRNAs were also shown to be significantly altered in AIS. In particular, members of the miR-30 family were upregulated in severe AIS and were associated with impaired osteogenic differentiation. Functional analyses demonstrated that extracellular vesicles enriched in these miRNAs reduced osteogenic marker expression and mineralization capacity, supporting a mechanistic link between circulating epigenetic factors and disease severity [63].

#### 3.2.3. Metabolic and Hormonal Biomarkers

Metabolic and hormonal alterations were consistently reported across studies. Elevated leptin levels were positively associated with curve severity, while reduced levels of osteocalcin and N-terminal telopeptide indicated impaired bone turnover [38].

Alterations in leptin signaling were also observed, with reduced free leptin index and increased soluble leptin receptor levels in AIS patients [50]. Increased ghrelin levels were associated with disease severity and osteopenia [53,57], while adiponectin levels were linked to reduced bone mineral density [54].

Metabolic enzymes were also involved. Reduced Dipeptidyl peptidase-4 (DPP-4) activity was observed in AIS and showed associations with metabolic parameters, although its relationship with curve severity was inconsistent [44,48].

In addition, mitochondrial and nuclear circulating DNA levels were altered in AIS, although their predictive value for disease progression remained limited [47].

#### 3.2.4. Bone Metabolism and Signaling Pathways

Markers of bone metabolism and osteogenesis were strongly associated with AIS severity. Reduced osteogenic activity, reflected by decreased RUNX2 expression and altered RANKL/OPG balance, was observed in more severe cases [53]. Increased osteoclast activity and bone resorption markers, such as TRAP5b, further supported a state of enhanced bone turnover [40]. Chen et al. [42] further supported the involvement of bone metabolism alterations in AIS. In this study, abnormal osteocyte lacuno-canalicular network structure was observed in AIS bone tissue, together with reduced canalicular number and length, increased lacunar volume and surface, and lower bone mechanical properties. In a parallel serological cohort, serum osteocalcin was negatively correlated with Cobb angle, supporting an association between impaired osteocyte function, altered bone formation, and curve severity.

At the signaling level, dysregulation of multiple pathways was reported. Activation of IL-6/STAT3 signaling was associated with cartilage degradation and increased MMP13 expression [52]. Similarly, Wnt/β-catenin signaling and associated regulators, including miR-145, were linked to impaired osteocyte function and disease severity [58].

Gene expression studies further highlighted alterations in pathways involved in extracellular matrix organization, muscle regulation, and inflammatory signaling, including Wnt-related genes and structural proteins [39]. These findings are consistent with evidence showing that epigenetic and extracellular vesicle-mediated mechanisms may directly impair osteogenic function and contribute to disease severity [63].

#### 3.2.5. Biomarkers Associated with Disease Progression

Only a limited number of studies directly assessed disease progression using longitudinal or follow-up data. Among these, lower circulating spermidine levels were associated with increased risk of progression, defined as a significant increase in Cobb angle or progression to severe curves [59]. Similarly, specific circulating miRNA panels demonstrated high predictive accuracy for progression to severe AIS, with some models achieving high sensitivity and specificity [37,55].

However, most studies relied on surrogate measures such as baseline Cobb angle or bone metabolic status to infer progression risk, rather than directly measuring longitudinal changes. Other investigations focused on disease presence, molecular mechanisms, or postoperative inflammatory responses without evaluating progression [47,56].

As a result, no biomarker has yet been consistently validated as a reliable predictor of AIS progression, highlighting the need for further longitudinal studies.

### 3.3. Risk of Bias Assessment

**Figure 2:**
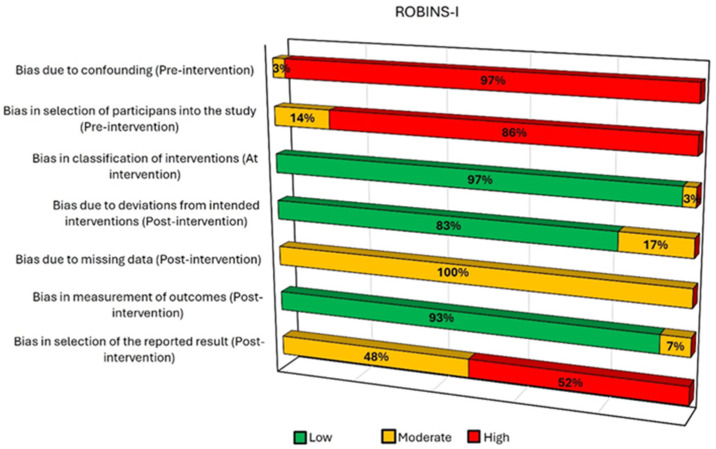
Risk of bias assessment of the included studies according to the ROBINS-I tool. The figure summarizes the proportion of studies rated as low, moderate, or serious risk of bias across the seven ROBINS-I domains, including confounding, participant selection, intervention classification, deviations from intended interventions, missing data, outcome measurement, and selection of reported results.

The risk of bias assessment using the ROBINS-I tool revealed substantial variability across domains (Figure 2). A high risk of bias was predominantly observed in pre-intervention domains, particularly due to confounding (97%) and selection of participants (86%), with only a small proportion of studies rated as moderate risk in these areas (respectively 3% and 14%). In contrast, most studies showed a low risk of bias in domains related to intervention classification (97%) and deviations from intended interventions (83%), with a minority rated as moderate (respectively 3% and 17%). Missing data was consistently rated as moderate risk across all studies (100%). Post-intervention domains generally demonstrated favorable assessments, with low risk of bias reported for outcome measurement (93%) and a small proportion of moderate risk (7%). However, bias in the selection of reported results remained a concern, with over half of the studies (52%) rated at high risk and the remainder (48%) at moderate risk. Overall, the findings indicate that while post-intervention methodological quality was generally acceptable, significant concerns persist in pre-intervention domains and selective reporting.

## 4. Discussion

**Figure 3:**
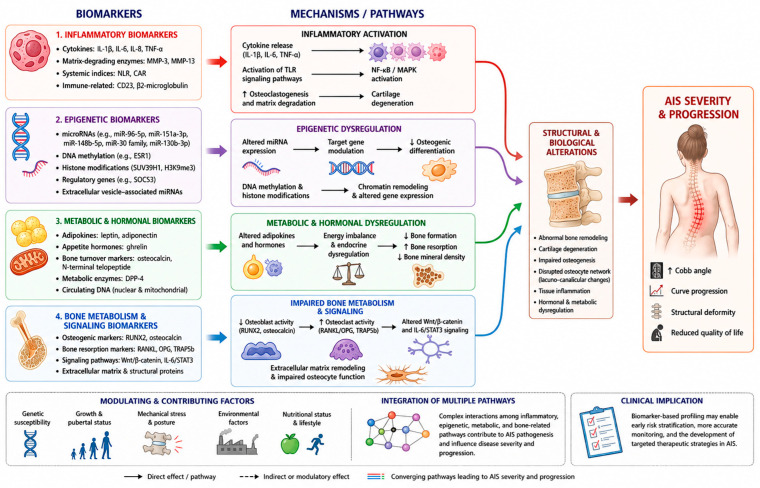
Schematic model illustrates circulating and tissue biomarkers, their underlying mechanisms, and their contribution to structural alterations and clinical outcomes in AIS, as identified in this review. The figure was created by the authors using Microsoft PowerPoint.

This systematic review provides a comprehensive synthesis of current evidence on circulating and tissue biomarkers associated with disease severity and progression in AIS. Overall, the findings support a multifactorial biological framework in which inflammatory, epigenetic, metabolic, and bone-related markers may be involved in biological processes associated with disease severity (Figure 3). However, only a limited subset of studies has evaluated their role in predicting disease progression. An additional challenge in interpreting progression-related biomarkers is the lack of standardization in progression definitions across studies. Thresholds for progression varied, commonly including increases of ≥5° or ≥10° in the major curve, and were often assessed over different follow-up durations. These differences are particularly relevant given the inherent variability of radiographic measurements and the influence of skeletal maturity, growth velocity, and treatment interventions on curve evolution. Consequently, heterogeneity in progression endpoints may have affected the comparability of findings and the identification of reliable predictive biomarkers.

In particular, a consistent association was observed between several biomarkers, particularly inflammatory cytokines [37,41,43,45,46,51,55,62,63], miRNAs [36,37,41,43,45,46,51,55,62,63], markers of bone metabolism [39,40,42,52,53,58,63], and curve severity, typically measured by the Cobb angle. These results support the hypothesis that AIS is not solely a structural deformity but also involves systemic biological alterations, including low-grade inflammation, dysregulated osteogenesis, and altered energy metabolism.

In contrast, evidence supporting their predictive value for longitudinal progression remains scarce [37,47,55,56,59]. This discrepancy reflects the predominance of cross-sectional study designs and represents a major limitation in the current literature.

The consistent association between inflammatory mediators and higher Cobb angles suggests a potential role of low-grade systemic inflammation in structural remodeling processes observed in AIS, particularly through cartilage degradation and extracellular matrix breakdown [64,65,66].

At the molecular level, the involvement of pathways such as TLR signaling and IL-6/STAT3 activation further strengthens the biological plausibility of inflammation-driven skeletal alterations [44,52,61]. These pathways are known to regulate osteoclastogenesis and tissue degeneration, suggesting that similar mechanisms may be involved in curve progression in AIS [67,68]. However, the distinction between disease-related inflammation and secondary responses remains critical, as postoperative elevations in inflammatory markers likely reflect acute physiological stress rather than intrinsic disease activity [56,69].

Similarly, epigenetic regulators, particularly circulating miRNAs, emerged as promising biomarkers, with several studies identifying specific signatures associated with disease severity [37,41,43,45,51,55,63] and, in some cases, progression risk [37,55].

Epigenetic regulation emerges as a particularly promising area, providing a potential link between genetic susceptibility and environmental influences. The identification of miRNA signatures associated with disease severity suggests that post-transcriptional regulation may play a key role in modulating skeletal growth and remodeling [70,71,72]. Notably, functional evidence from studies on extracellular vesicle-associated miRNAs indicates that these molecules have been shown in experimental models to influence osteogenic differentiation, providing biological plausibility for their association with AIS severity [73,74]. Nevertheless, the lack of external validation and the predominance of exploratory analyses currently limit their clinical applicability.

In parallel, metabolic and hormonal alterations, such as changes in leptin, ghrelin, and adipokines, highlight the interplay between energy balance and skeletal development in AIS [38,44,47,48,50,53,54,57]. This perspective aligns with emerging evidence linking AIS to broader metabolic phenotypes.

Bone metabolism markers further support this concept [39,40,42,52,53,58,63], suggesting that impaired osteogenic activity and increased bone resorption may be associated with curve progression [75,76]. Consistent evidence of altered bone metabolism reinforces the hypothesis of intrinsic skeletal fragility in AIS [77]. Indeed, impaired osteogenic activity, increased bone resorption, and structural abnormalities in the osteocyte network suggest that defective bone quality, rather than bone quantity alone, may be associated with greater disease severity. Dysregulation of key signaling pathways, including Wnt/β-catenin, provides a plausible mechanistic framework that may link molecular alterations to macroscopic deformity [78,79].

Despite growing interest in biomarker-based prediction, only a limited number of studies directly evaluated disease progression using longitudinal designs. Only a small subset of studies employed longitudinal designs, and among these, few identified biomarkers with potential prognostic value, such as specific miRNA panels or spermidine levels [37,55,59]. However, most studies relied on cross-sectional analyses, which, while useful for understanding biological mechanisms, do not allow causal inference or reliable prediction of disease trajectory. This represents a critical gap in the literature, as the clinical utility of biomarkers in AIS lies primarily in their ability to predict progression and guide early intervention.

The interpretation of these findings must also consider the methodological limitations identified through the risk of bias assessment. The ROBINS-I analysis revealed substantial concerns, particularly in pre-intervention domains. A high risk of bias due to confounding and participant selection was observed in most studies, reflecting the observational nature of the evidence and the frequent lack of adjustment for key clinical variables such as age, sex, skeletal maturity, and baseline curve severity. Furthermore, although ROBINS-I provided a standardized framework for assessing methodological quality across the heterogeneous study designs included in this review, it was originally developed for non-randomized intervention studies and may not fully capture all sources of bias relevant to observational biomarker research. Therefore, the risk-of-bias findings should be interpreted considering this methodological limitation. These factors are well-known determinants of AIS progression and may have influenced the reported associations, thereby limiting the internal validity of the findings.

In contrast, domains related to intervention classification and post-intervention processes generally showed lower risk of bias, suggesting that biomarker measurement and outcome assessment were relatively consistent and methodologically sound across studies. However, the presence of a moderate risk of bias due to missing data in all studies indicates potential issues with incomplete datasets and follow-up, which may affect the robustness of the results. Furthermore, the high proportion of studies with a serious risk of bias in the selection of reported results raises concerns about selective reporting and publication bias, potentially leading to an overrepresentation of statistically significant findings.

To provide a more structured synthesis of the available evidence, biomarker categories were classified according to the consistency and replication of findings reported across the included studies (Table 7). Overall, inflammatory cytokines, miRNAs, and bone metabolism markers showed the most consistent associations with AIS severity, whereas evidence for progression-related biomarkers remains limited and largely exploratory. Most candidate biomarkers still require independent validation before they can be considered clinically useful predictors of disease progression.

Several limitations should be considered when interpreting these findings. First, the predominance of cross-sectional designs limits the ability to establish causal relationships or predictive value. Second, substantial heterogeneity was observed in study design, patient populations, biomarker selection, and analytical methods. Third, sample sizes were often small, particularly in translational and tissue-based studies, reducing statistical power. Finally, differences in outcome definitions, especially regarding disease progression, further limit comparability across studies.

The identification of reliable biomarkers for AIS progression remains a key unmet clinical need. An additional emerging area of interest involves biomarkers of skeletal growth and maturity. Although not included in the present systematic review because they did not meet the predefined eligibility criteria, recent studies have investigated circulating biomarkers associated with residual growth potential rather than curve severity or progression. For example, Welborn et al. identified Collagen X Biomarker (CXM) as a promising indicator of longitudinal bone growth, demonstrating strong correlations with established measures of skeletal maturity, including Sanders score, Risser stage, bone age, and peak height velocity [80]. Such biomarkers may complement traditional radiographic assessments and could potentially contribute to more individualized prediction of progression risk in AIS. However, further studies are needed to clarify their direct relationship with curve progression and clinical outcomes. Ideally, such biomarkers should be measurable in accessible biological samples, reflect underlying disease mechanisms, and enable early and accurate risk stratification.

Future research should prioritize large-scale, longitudinal studies integrating multi-omics approaches, including transcriptomics, metabolomics, and proteomics. Standardization of study design, biomarker assessment, and outcome definitions will be essential to facilitate comparison across studies and enable clinical translation. In addition, combining biomarkers with clinical and radiographic parameters may improve predictive models and support personalized management strategies in AIS.

## 5. Conclusions

Taken together, the current evidence suggests that multiple circulating and tissue biomarkers are associated with AIS severity, but their role in predicting disease progression remains insufficiently established. Future research should prioritize well-designed prospective longitudinal studies with standardized definitions of progression, adequate control of confounding variables, and rigorous methodological approaches. The integration of multi-omics strategies and advanced analytical models may further improve the identification of robust biomarker signatures. Ultimately, improving study quality and reducing bias will be essential to translate biomarker research into clinically useful tools for early risk stratification and personalized management of AIS.

## Figures and Tables

**Table 1:** Study design and demographic characteristics of the included studies.

Reference.	Country	Study Design	Sample Size (AIS/Controls)	Mean Age (±SD)	Sex	AIS Type	Follow-Up
[35]	Canada	Cross-sectional histological study	20 AIS/6 Controls	AIS: 15.23 ± 2.36; Controls: 34.33 ± 13.31	75% Female (AIS)	Lenke types 1–6	None
[36]	Poland	Cross-sectional (epigenetic tissue study)	29 AIS	14.5 ± 1.5	Female	Surgical AIS	None
[37]	Canada	Prospective cross-sectional with longitudinal follow-up	116 AIS/~30 Controls	13.3 ± 1.7	Female and Male	Severe (≥45°), Moderate (25–44°), Non-progressive (<15°)	Until skeletal maturity
[38]	Poland	Cross-sectional study	77 AIS	14.7 ± 2.17	Female	AIS	None
[39]	Italy	Observational cross-sectional (tissue-based exploratory study)	21 AIS/6 Controls	AIS: 18 ± 3.7; Controls: 63 ± 11.0	AIS: 13 F/8 M	Progressive AIS (>40°, surgical)	None
[40]	Japan	Cross-sectional study	33 AIS	14.7 (11–19)	29 F/4 M	Not specified	None
[41]	China	Cross-sectional study	20 AIS/20 Controls	AIS: 16 ± 1.0; Controls: 16.2 ± 1.1	AIS: 15 F/5 M; Controls: 13 F/7 M	Not specified	None
[42]	China	Case–control study	99 AIS/31 Controls	AIS: 15.2 ± 2.0; Controls: 14.3 ± 1.1	Female	AIS	None
[43]	China/Canada	Case–control + microarray study	Discovery: 4 AIS/4 Controls; Validation: 100 AIS/52 Controls	~14–15 years (validation)	Female	AIS	None
[44]	China	Case–control + experimental study	Serum: 80 AIS/50 Controls; Muscle: 45 AIS/30 Controls	Serum: AIS 13.9 ± 2.5/Controls 14.3 ± 3.3; Tissue: AIS 15.4 ± 2.7/Controls 15.8 ± 4.2	Female	Single thoracic AIS	None
[45]	Spain	Prospective case–control study	Discovery: 17 AIS/10 Controls; Validation: 30 AIS/17 Controls; Independent: 17 AIS/7 Controls	~14–15 ± 2	Predominantly Female (~5:1)	AIS	≥2-year
[46]	China	Case–control (tissue study)	11 AIS/10 Controls	AIS: 16.86 ± 1.86; Controls: 19.80 ± 4.57	AIS: 5 M/6 F; Controls: 6 M/4 F	AIS	None
[47]	China	Case–control study	69 AIS/21 Controls	AIS: 14.4 ± 0.3; Controls: 13.8 ± 0.9	AIS: 52 F/17 M; Controls: 15 F/6 M	Not specified	None
[48]	Canada	Case–control study	113 AIS/62 Controls	AIS: 13.7 ± 1.4; Controls: 14.3 ± 1.5	Female	AIS	None
[49]	China	Case–control study	56 AIS/10 Controls	AIS: 13.6 ± 2.5; Controls: 12.2 ± 2.8	AIS: 6 F/4 M; Controls: 32 F/24 M	Not specified	None
[50]	China	Case–control study	148 AIS/116 Controls	AIS: 12.9 ± 0.6; Controls: 13.0 ± 0.5	Female	Not specified	None
[51]	China	Case–control study	90 AIS/45 Controls	AIS: 13.1 ± 1.8; Controls: 11.9 ± 2.4	Female	Not specified	None
[52]	China	Case–control (cross-sectional + experimental)	Serum: 161 AIS/140 Controls; Cartilage: 18 AIS/14 Controls; ELISA: 48 AIS/40 Controls	AIS: 12.6 ± 3.5; Controls: 11.2 ± 4.2	Female	AIS	None
[53]	China	Case–control study	563 AIS/281 Controls; Subgroups: 83 AIS (osteopenia)/44 Controls	~14–18 years	Predominantly Female	AIS (osteopenia subgroup defined by Z-score < −1)	None
[54]	China	Case–control study	92 AIS/35 Controls	AIS: 13.9 ± 2.2; Controls: 14.3 ± 2.0	AIS: 45 F/47 M; Controls: 11 F/24 M	Not specified	None
[55]	Czech Republic	Prospective monocentric biomarker study	114 AIS/89 Controls	AIS: 12.4 ± 0.6; Controls: 12.0 ± 0.6	AIS: 88% Female; Controls: 74% Female	Juvenile (43%)/Adolescent (57%) AIS	24 months
[56]	Italy	Prospective pilot study	30 AIS (PSF surgery)	15.3 ± 1.8	26 F/4 M	Not specified	2 days post-surgery
[57]	China	Prospective observational study	105 AIS/40 Controls	AIS: 12.4 ± 1.9; Controls: 12.8 ± 1.2	Female	Not specified	18 months
[58]	China (Hong Kong)	Translational case–control study	Bone: 13 AIS/10 Controls; Serum: 74 AIS	AIS: 15.54 ± 1.76; Controls: 15.60 ± 5.77	Not reported	AIS	None
[59]	China	Translational (clinical + animal + in vitro)	28 AIS (muscle); 27 AIS (serum)	Serum cohort: 10–13.5 years	Female	Mild and severe progressive thoracic AIS	Up to 6 years (serum cohort)
[60]	Turkey	Multicenter case–control study	419 AIS/381 Controls	14.0 ± 2.0	AIS: 257 F/162 M; Controls: 234 F/147 M	Not specified	None
[61]	Canada	Experimental + observational tissue study	35 AIS/16 Controls	AIS: 16.2 ± 2.6; Controls: 27.6 ± 7.4	AIS: 78% Female	Not specified (Lenke classification mentioned)	None
[62]	China	Genetic association study	Genotyping: 476 AIS/672 Controls; Expression: 53 AIS/41 Controls	AIS: 14.3 ± 1.7; Controls: 14.8 ± 1.9	Mixed	Lumbar AIS	Not reported
[63]	Italy	Observational clinical proof-of-concept study	20 AIS/10 Controls	AIS: 14.7 ± 1.5; Controls: 15 ± 2.3	AIS: 17 F/3 M; Controls: 5 F/5 M	Idiopathic scoliosis (Lenke classification)	≥2 years

Abbreviations = AIS: Adolescent Idiopathic Scoliosis; SD: Standard Deviation; F: Female; M: Male; ELISA: Enzyme-Linked Immunosorbent Assay; PSF: Posterior Spinal Fusion.

**Table 2:** Clinical characteristics of study populations included in the review.

Reference	BMI (kg/m^2^)	Body Characteristics	Skeletal Maturity (Risser)	Pubertal Stage	Other Relevant Variables
[35]	Not reported	Not reported	Not reported	Not reported	Cobb angle: ~45–100°; facet joint asymmetry; Lenke classification; level-specific sampling
[36]	Not reported	Not reported	Median ~4 (no group differences)	Not reported	Cobb angle: 52–115°; subgroup analysis (≤70° vs. >70°); convex vs. concave muscle
[37]	Not reported	Not reported	Risser 0–2 (baseline); 4–5 (follow-up)	Pre-menarche or <1-year post-menarche	Cobb angle stratification; sex-specific analyses
[38]	18.38 ± 2.56	FM, FFM, PMM, TBW	Not reported	Tanner stage (adjusted)	WHtR associated with severity
[39]	AIS: ~17–28	Not reported	Risser 2–5	Menarche status (females)	Cobb angle: 45–86°; curve localization; convex vs. concave tissue comparison
[40]	BMI Z-score: −0.3 (range −1.8 to 2.2)	Weight, BFM, % BF, FFM, SMM, right and trunk LM	Risser distribution: Grade 1 (*n* = 2), Grade 2 (*n* = 4), Grade 3 (*n* = 8), Grade 4 (*n* = 13), Grade 5 (*n* = 6)	Not reported	Not reported
[41]	AIS: 16.7 ± 2.4; Controls: 18.4 ± 2.5	BMD	AIS: 2.7 ± 0.8; Controls: 2.7 ± 1.0	Not reported	BMD inversely associated with AIS
[42]	AIS: 18.2 ± 2.1; Controls: 20.2 ± 3.0	Not reported	Not reported	Tanner stage: AIS 2.8 ± 1.5; Controls: 3.2 ± 1.9	Not reported
[43]	Not reported	Height, weight, arm span, sitting height	Not explicitly reported	Tanner stage (breast and pubic hair)	Lower body weight and femoral neck BMD in AIS; altered HR-pQCT parameters; menarche recorded
[44]	AIS: 17.9 ± 2.3; Controls: 18.5 ± 3.0	Muscle density: 0.84 ± 0.10 g/cm^2^ (AIS)	Not reported	Comparable age, weight, height	Lower BMI associated with reduced DPP-4 expression; impaired insulin sensitivity suggested
[45]	AIS: ~19.8 ± 3.0	Not reported	2.46–3.83 ± ~1.8–1.9	Menarche status recorded	Cobb angle: 10–>40°; ~40% positive family history; SF-36 assessed
[46]	Not reported	Not reported	Not reported	Not reported	Mean Cobb angle: 45.6°; facet joint cartilage samples collected during surgery
[47]	Not reported	Height, weight	Not reported	Not reported	Not reported
[48]	AIS: 19.5 ± 3.7; Controls: Not reported	FM, LM, BMD	Not reported	Not reported	Not reported
[49]	AIS: 20.1 ± 1.5; Controls: 20.1 ± 2.13	Not reported	AIS: 2.5 ± 1.8; Controls: 2.0 ± 2.1	Not reported	Not reported
[50]	AIS: 17.6 ± 2.1; Controls: 18.4 ± 2.2	Height, weight, BFM, BMI, % BF, FFM, SMM, right and trunk LM	Not reported	Tanner stage: breast (AIS 3.0 ± 0.8; Controls 3.1 ± 0.7); pubic hair (AIS 2.5 ± 0.8; Controls 2.5 ± 0.9)	Body composition variables inversely associated with AIS
[51]	AIS: 17.9 ± 1.1; Controls: 17.5 ± 1.2	FM, LM	Not reported	Not reported	Not reported
[52]	Not reported	Not reported	Not reported	Not reported	Cobb angle: 37.5 ± 12.4° (AIS); age-matched controls
[53]	AIS: 17.76 ± 2.60; Controls: 20.87 ± 4.54	BMD (LSBMD, FNBMD), Z-score, weight, height	AIS: 2.12 ± 1.06; Controls: 2.32 ± 0.96	Not reported	Lower BMI and BMD; osteopenia defined as Z-score < −1
[54]	AIS: 17.3 ± 1.1; Controls: 18.8 ± 1.2	Not reported	AIS: 2.0 ± 1.7; Controls: 2.3 ± 1.7	Not reported	Not reported
[55]	AIS: 17.8 ± 0.6; Controls: 18.4 ± 0.5	Not reported	Risser 0–3	Menarche status (pre-/post-)	Cobb angle: baseline 24.8°, final 29.6°; risk groups (low ≤ 25°, moderate 25–35°, high ≥ 35°); brace treatment
[56]	21.6 ± 4.4	Not reported	Not reported	Not reported	Not reported
[57]	AIS: 17.5 ± 1.4; Controls: 18.2 ± 1.3	FM, LM	AIS: 2.0 ± 1.8; Controls: 2.4 ± 1.6	Not reported	Menstrual status, cBMI, and age associated with severity
[58]	AIS: 18.24 ± 2.53; Controls: 17.88 ± 2.70	Not reported	AIS: 4.23 ± 0.44; Controls: 3.80 ± 1.30	Tanner stage: AIS 3.31 ± 1.03; Controls 3.20 ± 1.64	Cobb angle: bone cohort 58.15 ± 11.41°; serum cohort 42.11 ± 23.72°
[59]	Not reported	Not reported	Not reported (skeletally immature)	Early adolescence (10–13.5 years)	Curve progression (>6° increase or Cobb > 40°); paraspinal muscle asymmetry; focus on LBX1
[60]	Not reported	Height, weight	Not reported	Not reported	Not reported
[61]	Not reported	Not reported	Not reported	Not reported	Cobb angle; facet joint OA grade; intervertebral rotation; 3D EOS imaging
[62]	Not reported	Not reported	Adolescents (10–18 years)	Not reported	Lumbar curve > 20° (thoracic < 10°); convex vs. concave muscle sampling
[63]	21.6 ± 4.5	Not detailed	3.4 ± 1.8	Not explicitly reported	Cobb angle: 54.6° (range 21–92); Lenke classification; predominantly severe AIS

Abbreviations = AIS: Adolescent Idiopathic Scoliosis; BMI: Body Mass Index; FM: Fat Mass; FFM: Fat-Free Mass; PMM: Predicted Muscle Mass; TBW: Total Body Water; WHtR: Waist-to-Height Ratio; vs.: versus; LBX1: Ladybird Homeobox 1; BMD: Bone Mineral Density; HR-pQCT: High-Resolution Peripheral Quantitative Computed Tomography; DPP-4: Dipeptidyl Peptidase-4; LM: Lean Mass; OA: Osteoarthritis; EOS: Low-dose biplanar X-ray imaging system; 3D: three-dimensional; LSBMD: Lumbar Spine Bone Mineral Density; FNBMD: Femoral Neck Bone Mineral Density; SMM: Skeletal Muscle Mass; BFM: Body Fat Mass; cBMI: Corrected Body Mass Index.

**Table 3:** Summary of inflammatory and metabolic biomarkers associated with disease severity and progression in AIS.

Reference	Biomarker	Category	Biological Sample	Measurement Method	Direction (↑/↓)	Statistical Significance
[35]	IL-1β	Inflammatory cytokine	Facet joint tissue	IHC	↑ in AIS	*p* < 0.001
	IL-6	Cytokine	Tissue		No change	Not significant
	MMP-3	Matrix metalloproteinase			↑ in AIS	*p* < 0.001
	MMP-13				↑ in AIS	
	Proteoglycans	ECM component	Cartilage	Histology (Safranin-O)/MATLAB 26.1 quantification	↓ in AIS	*p* < 0.0001
	SLRPs (decorin, chondroadherin)	ECM proteins	Tissue	WB	↑ fragmentation	Severity-dependent
[36]	ESR1 T-DMR1 methylation	Epigenetic marker	Paraspinal muscle	Pyrosequencing	↑ superficial vs. deep muscle	*p* < 0.01
	ESR1 T-DMR2 methylation					*p* < 0.05
	ESR1 expression	Gene expression	Muscle	qPCR	No difference	Not significant
	ESR1 T-DMR2 (concave side)	Epigenetic marker	Deep paravertebral muscle	Pyrosequencing	↑ with severity (Cobb > 70°)	*p* < 0.05
[37]	Multiple circulating miRNAs panel (let-7f-5p, miR-1-3p, miR-18a-3p, miR-19a-3p, miR-19b-3p, miR-103a-3p, miR-107, miR-133b, miR-143-3p, miR-148a/b-3p, miR-152-3p, miR-214-3p, miR-551b-3p, miR-576-5p)	Epigenetic biomarkers	Plasma	Microarray + RT-qPCR	↑ in severe AIS	*p* < 0.05
[38]	Leptin	Hormone/metabolic marker	Serum	ELISA	↑ with severity	*p* < 0.01
	OC	Bone turnover marker		EIA	↓ with severity	*p* < 0.05
	NTx	Bone resorption marker		ELISA		*p* < 0.01
[39]	PCDH10	Epigenetic genes	Bone, muscle, ligament, blood	RT-qPCR	↓ in AIS	*p* < 0.05
	FBN2, CRTC1		Bone			
	FRZB	Wnt pathway gene	Muscle			
	LRP6					*p* < 0.01
	MSTN	Muscle regulator				*p* < 0.05
	WNT1	Wnt pathway gene			↑ in AIS	
	WNT10					*p* < 0.01
	FBN1	ECM-related gene	Ligament			
	miR-145	miRNA	Bone, blood		↓ in AIS	*p* < 0.05
	miR-675		Bone			
[40]	TRAP5b	Bone resorption marker	Serum	ELISA	↑ in AIS	*p* = 0.032
[41]	miRNA-130b-3p	miRNA	Plasma	RT-qPCR	↑ in AIS	*p* < 0.0001
[42]	OCN	Bone formation marker	Serum	ELISA	↑ in AIS	*p* < 0.05
	P1NP					
	CTX	Bone resorption marker			↓ in AIS	
	OPN	Bone matrix protein			Not clearly specified	Not reported
	DKK1	Wnt pathway inhibitor			↑ in AIS	*p* < 0.05
	Sclerostin	Bone metabolism regulator			Not clearly specified	Not reported
[43]	miR-96-5p	Epigenetic regulator (miRNA)	Bone/plasma	Microarray (bone) + TaqMan RT-qPCR (plasma)	↑ in AIS	*p* = 0.001
[44]	DPP-4	Metabolic enzyme	Serum/paraspinal muscle	ELISA (serum); RT-qPCR; WB (tissue)	↓ in AIS (serum ~0.76 fold; tissue ~0.68 fold)	*p* < 0.05
	STAT1	Transcription factor	Paraspinal muscle	RT-qPCR, WB	↓ in AIS	
[45]	miR-122-5p, miR-27a-5p, miR-223-5p, miR-1306-3p	miRNAs	Plasma	NGS + RT-qPCR	↑ in AIS	*p* < 0.05
	miR-671-5p				Variable	Not significant
[46]	H3K9me3	Histone methylation marker	Chondrocytes	WB/ChIP	↑ in AIS	*p* < 0.05
	SUV39H1	Histone methyltransferase		RT-qPCR; WB		
	miR-15a	miRNA		RT-qPCR	↓ in AIS	
	Bcl2	Anti-apoptotic protein		RT-qPCR; WB	↑ in AIS	
	PCNA	Proliferation marker	Tissue	IF		
	Collagen II (COL2A1)	Cartilage markers	Chondrocytes	RT-qPCR		
	Collagen X (COL10A1)				↓ in AIS	
[47]	Circulating ccf-nDNA	Circulating biomarker	Plasma	qPCR	↓ in AIS; ccf n-DNA levels for GAPDH (*p* = 0.027) and for ACTB (*p* = 0.030)	*p* < 0.05
[48]	DPP-4 activity	Metabolic regulator	Serum	ELISA	↓ in AIS	*p* = 0.0357
[49]	CD23, B2M	Immune markers	Serum	ELISA	↓ in AIS	*p* < 0.0001
	FAP	ECM remodeling				
[50]	Leprin	Hormone	Serum	ELISA	↓ in AIS	*p* = 0.013
	FLI	Metabolic marker				*p* = 0.002
	sOB-R	Hormone receptor			↑ in AIS	
[51]	miR-941, miR-151a-3p, miR-148b-5p	miRNAs	Plasma	RT-qPCR	↑ in AIS	*p* < 0.05
[52]	Androgens (DHT/testosterone)	Hormonal	Serum	ELISA	↑ Prothrombin activity INR post-op	*p* < 0.0005
	IL-6	Inflammatory cytokine	Cartilage/serum/cell culture	ELISA, WB, qPCR	↑ aPTT ratio post-op	
	MMP-13	Cartilage degradation marker	Cartilage/cells	WB	↑ PCR post-op	
	AR	Androgen receptor	Cartilage	WB, IHC	↑ Glucose post-op	
	IL-1α, IL-1β, IL6, IL8, IL10, TNF-α, PGE	Inflammatory parameters	Serum	ELISA	↑ IL6 in post-op period	*p* < 0.05
[53]	Ghrelin	Hormone	Plasma	ELISA	↑ in AIS osteopenia	*p* < 0.01
	RANKL	Osteoclastogenic marker	Bone (cancellous)	qPCR/WB	↑ in AIS	*p* < 0.05
	OPG	Bone protective marker			Slight impairment in response	Not significant/context-dependent
	RANKL/OPG ratio	Bone remodeling	Bone		↑ in AIS	*p* < 0.05
	RUNX2	Osteogenic marker		qPCR	↓ in AIS	
	Osteoclast number	Cellular marker	Facet joint	TRAP staining	↑ in AIS	*p* < 0.01
[54]	Adiponectin	Metabolic hormone	Serum	ELISA	↑ in AIS	*p* < 0.01
[55]	Circulating miRNAs (48 diagnostic, 7 prognostic)	Epigenetic biomarkers	Plasma	Small RNA sequencing (NGS), DESeq2 analysis	Mixed ↑/↓	*p* < 0.05
	miR-4451	Prognostic miRNA		NGS	↓ in high-risk AIS	*p* < 0.01
[56]	Hematologic parameters (WBC count, RBC count, Hemoglobin, Hematocrit, MCV, MCH, MCHC, RDW, Basophils, Neutrophils, Lymphocytes, Monocytes, Eosinophils, Platelet Count, MPV, Prothrombin Activity Ratio, Prothrombin Activity, aPTT, Glucose, Creatinine, CRP)	Blood biomarkers	Blood	Clinical assays	Pre-op ↓; Post-op ↑ inflammatory markers	*p* < 0.0005
[57]	Ghrelin	Hormone	Serum	ELISA	↑ in AIS	*p* < 0.01
	Leptin				↓ in AIS	*p* < 0.05
[58]	miR-145-5p	miRNA	Bone/plasma	RT-qPCR	↑ in AIS	*p* < 0.05 (bone); Not significant (plasma)
	β-catenin (CTNNB1)	Wnt signaling	Bone, osteoblasts, osteocytes	qPCR/WB		*p* < 0.05
	SOST	Osteocyte marker	Osteocytes, serum	ELISA/qPCR	↓ in AIS	
	OPG	Bone marker	Serum/cells			
	OPN		Serum	ELISA		
	DMP1, FGF23	Bone metabolism	Bone/osteocytes	qPCR		
[59]	Spermidine	Metabolic (polyamine)	Serum	Metabolomics	↓ in progressive AIS (hematocrit pre-op)	*p* < 0.0005
	ODC1	Enzyme (polyamine)	Muscle	WB/qPCR	↓ in AIS (lymphocytes pre-op)	
	SAT1				↓ in AIS (eosinophils pre-op)	
	LBX1	Genetic regulator		qPCR	↓ Basophils pre-op	*p* < 0.005
[60]	CRP, WBC, neutrophils, lymphocytes, monocytes, NLR, CAR, phosphorus	Inflammatory biomarkers	Blood	Hematology + biochemistry	↑ in AIS	*p* < 0.01
	Calcium, platelet count, MCV, PLR	Metabolic markers		Biochemistry	↓ in AIS	
[61]	TLR pathway activation	Inflammatory pathway	Facet joint chondrocytes	RNA-seq/qPCR/ELISA	↓ Platelet counts pre-op	*p* < 0.0005
	M-CSF	Cytokines	Tissue/conditioned media	qPCR/ELISA	↓ Creatinine pre-op	*p* < 0.05
	GM-CSF				↑ WBC post-op	*p* < 0.0005
	IL-1α, IL-6, IL-8, TNF-α			RNA-seq/ELISA	↑ Monocytes post-op	
	CXCL-1, CXCL-10	Chemokines			↑ Neutrophils post-op	
	RANKL	Bone remodeling marker	Tissue	qPCR	↑ MPV post-op	*p* < 0.05
	OPG			qPCR/ELISA	↑ Prothrombin activity ratio post-op	*p* < 0.0005
[62]	SOCS3 expression	Inflammatory regulator	Paravertebral muscle	RT-qPCR	↓ in AIS	*p* < 0.05
	SOCS3 rs4969198 (GG genotype)	Genetic variant	Blood DNA	PCR genotyping	↑ risk allele frequency	*p* = 0.000
	SOCS3 protein (inferred via mRNA)	Inflammatory regulator	Muscle	RT-qPCR	↓ in AIS severity groups	*p* < 0.01
[63]	miR-30 family (miR-30a-5p, miR-30d-5p, miR-30a-3p, miR-30e-3p)	miRNAs	Plasma/EVs	Microfluidic RT-qPCR arrays	↑ in severe AIS	*p* < 0.05
	miR-1294, miR-200a, miR-548m		Plasma	RT-qPCR	↓ in AIS	
	RUNX2, ALPL, COL1A1	Osteogenic markers	hMSCs (after EV treatment)	RT-qPCR/ELISA		
	SAA1, CFL1 (EV proteins)	EV proteins	Plasma EVs	LC-MS/MS proteomics	↑ in AIS	
	Osteogenic mineralization	Functional outcome	hMSCs	Alizarin Red staining	↓ in AIS	qualitative + significant

Abbreviations = AIS: Adolescent Idiopathic Scoliosis; ELISA: Enzyme-Linked Immunosorbent Assay; EIA: Enzyme Immunoassay; RT-qPCR: Reverse Transcription Quantitative Polymerase Chain Reaction; WB: Western Blot; NGS: Next-Generation Sequencing; ChIP: Chromatin Immunoprecipitation; IHC: Immunohistochemistry; miRNA: microRNA; MMP: Matrix Metalloproteinase; ECM: Extracellular Matrix; SLRPs: Small Leucine-Rich Proteoglycans; DPP-4: Dipeptidyl Peptidase-4; STAT: Signal Transducer and Activator of Transcription; TLR: Toll-Like Receptor; RANKL: Receptor Activator of Nuclear Factor κB Ligand; TNF-α: Tumor Necrosis Factor Alpha; RUNX2: Runt-Related Transcription Factor 2; OPG: Osteoprotegerin; EVs: Extracellular Vesicles; TRAP: Tartrate-Resistant Acid Phosphatase; hMSCs: Human Mesenchymal Stem Cells; OC: osteocalcin; LBX1: Ladybird Homeobox 1; ODC1/SAT1: Polyamine metabolism enzymes; IL: Interleukin; COL1A1/COL2A1/COL10A1: Collagen Type I/II/X; IF: Immunofluorescence; M-CSF/GM-CSF: Macrophage/Granulocyte-Macrophage Colony-Stimulating Factor; CXCL: C-X-C Motif Chemokine Ligand; AR: Androgen Receptor; DHT: Dihydrotestosterone; SOST: Sclerostin; DMP1: Dentin Matrix Protein 1; FGF23: Fibroblast Growth Factor 23; OPN: Osteopontin; ALPL: Alkaline Phosphatase; SAA1: Serum Amyloid A1; CFL1: Cofilin-1; ↓: decrease; ↑: increase.

**Table 4:** Association between inflammatory biomarkers and curve severity (Cobb Angle) in patients with AIS.

Reference	Clinical Outcome	Statistical Method	Associated Biomarkers	Direction of Association	Notes
[35]	Cobb angle	Histology, IHC, WB (comparative analysis)	MMP-3, MMP-13, IL-1β, proteoglycan loss, SLRP fragmentation	Positive	Tissue degeneration increases with severity; SLRP fragmentation mainly >70°
[36]	Cobb angle	Spearman/Pearson correlation	ESR1 T-DMR2 methylation (CpG2, CpG6)	Positive	Significant on concave side only
	Cobb angle (≤70° vs. >70°)	Group comparison	ESR1 T-DMR2 methylation		Higher methylation in severe curves
[37]	Cobb angle categories (≤25°, 25–44°, ≥45°)	Multivariate regression + RFM	miR-1-3p, miR-19a-3p, miR-19b-3p, miR-133b, miR-143-3p, miR-148b-3p	Positive	Strong association with severe AIS; AUC = 1.00
[38]	Cobb angle	Multivariate regression	Leptin	Positive	Higher leptin associated with greater severity
			OC, NTx	Negative	Lower bone turnover associated with greater severity
[39]	Cobb angle	Correlation analysis	Multiple genes	Mixed	No consistent biomarker pattern; small sample size
[40]	Cobb angle (mean 52°)	Not reported	Not reported	Not reported	Not reported
[41]	Not reported	Not reported	miR-130b-3p	Positive	Higher levels associated with greater severity
[42]	Cobb angle (mild vs. severe AIS): 26.6 ± 9.1° vs. 65.8 ± 14.1°	Pearson correlation	OCN	Negative	Lower OCN associated with greater severity (*p* = 0.003)
[43]	Cobb angle	Multivariate logistic regression	miR-96-5p	Positive	Independent predictor of severity
[44]	Cobb angle	Pearson correlation	DPP-4	None	No significant association (r = −0.17, *p* = 0.27)
[45]	Cobb angle	RFM + regression + ROC analysis	miR-122-5p, miR-27a-5p, miR-223-5p, miR-1306-3p	Positive	Signature predicts severity (AUC = 0.95; sensitivity 92.9%; specificity 72.7%)
[46]	Cobb angle	Histological + molecular analysis	SUV39H1, H3K9me3, miR-15a, Bcl-2	Positive (indirect)	Epigenetic repression linked to severity via chondrocyte proliferation
[47]	Cobb angle (36.1 ± 3.3°)	Not reported	Not reported	Not reported	Not reported
[48]	Cobb angle (33° ± 15°)	Not reported	DPP-4 activity	Negative	Lower DPP-4 activity associated with greater severity
[49]	Cobb angle categories (10–20°, 20–40°, >40°)	Multiple linear regression	FAP, CD23	Negative	Lower levels associated with greater severity
[50]	Cobb angle (23.6 ± 9°)	Multiple regression	Leptin, FLI	Negative	Lower leptin associated with greater severity
			sOB-R	Positive	Higher sOB-R associated with severity
[51]	Cobb angle (mild vs. severe AIS): 24.4° ± 6.3° vs. 63° ± 13.1°	Not reported	miR-941, miR-151a-3p, miR-148b-5p	Positive	Higher miRNAs associated with severe AIS
[52]	Cobb angle	Correlation + regression	Androgens (DHT/testosterone), IL-6, AR	Mixed	Androgens ↓ (negative); IL-6 ↑ (positive)
[53]	Osteopenia/inferred severity	*t*-test + regression	Ghrelin	Positive	Higher ghrelin associated with severity
	Bone loss severity	Molecular analysis	RANKL/OPG ratio		Higher ratio associated with worse bone status
	Osteogenic activity	Expression analysis	RUNX2	Negative	Lower RUNX2 associated with severity
[54]	Cobb angle (22.8 ± 7°)	Not reported	Not reported	Not reported	Not reported
[55]	Cobb angle (risk stratification)	Logistic regression + ROC analysis	7-miRNA signature; miR-4451	Mixed	Higher risk score → higher severity; miR-4451 inversely associated (AUC = 0.83)
[56]	Lumbar Cobb angle (51.8° ± 12.9°, pre-op; 16.5° ± 3.8°, post-op). Thoracic Cobb angle (61.8° ± 14.6°, pre-op; 14.3° ± 7.2°, post-op)	Not reported	Not reported	Not reported	Not reported
[57]	Cobb angle (progressive vs. stable): 28.9° ± 13.8° vs. 21.6° ± 6.4°	Multivariate logistic regression	Ghrelin	Positive	Higher ghrelin associated with progression/severity
[58]	Cobb angle	Correlation + regression	miR-145, CTNNB1 (β-catenin)	Positive (indirect)	miR-145 linked to Wnt signaling activation
[59]	Cobb angle	Spearman correlation	LBX1 (concave/convex ratio)	Negative	Lower ratio associated with higher severity
	Cobb angle (>40° progressive AIS)	Group comparison	Spermidine		Lower levels in severe progressive AIS
[60]	Cobb angle (11–20°; 65°)	Correlation analysis	CRP, NLR, CAR	Positive	Higher inflammatory indices associated with greater severity
[61]	OA severity/vertebral rotation	Correlation + regression	M-CSF	Positive	Higher M-CSF linked to degeneration
	Cobb angle (indirect)	Not explicitly modeled	TLR2/4 pathway, M-CSF	Positive (trend)	Inflammatory activation linked to severity
[62]	Cobb angle	Pearson correlation	SOCS3 expression	Negative	Lower SOCS3 associated with greater severity (r = −0.472)
		Group comparison	SOCS3 rs4969198 (GG genotype)	Positive	GG genotype associated with larger curves
[63]	Cobb angle	Differential expression + bioinformatics	miR-30 family (miR-30a/d/e)	Positive	Associated with severe female AIS
	Cobb angle (severity groups)	Group comparison	EV-derived miR-30 cluster	Positive	Not observed in mild/moderate AIS or males

Abbreviations = AIS: Adolescent Idiopathic Scoliosis; OC: Osteocalcin; NTx: N-terminal telopeptide; ESR1: Estrogen Receptor 1; LBX1: Ladybird Homeobox 1; RFM: Random Forest Model; miRNA (miR): microRNA; AUC: Area Under the Curve; ROC: Receiver Operating Characteristic; IHC: immunohistochemistry; WB: Western blot; MMP: Matrix Metalloproteinase; SLRP: Small Leucine-Rich Proteoglycans; DPP-4: Dipeptidyl Peptidase-4; SOCS3: Suppressor of Cytokine Signaling 3; OA: Osteoarthritis; M-CSF: Macrophage Colony-Stimulating Factor; TLR: Toll-Like Receptor; IL-1β/IL-6: Interleukin-1 beta/Interleukin-6; DHT: Dihydrotestosterone; AR: Androgen Receptor; OPG: Osteoprotegerin; RANKL: Receptor Activator of Nuclear Factor κB Ligand; RUNX2: Runt-related transcription factor 2; CTNNB1: β-catenin gene; CRP: C-reactive protein; NLR: Neutrophil-to-Lymphocyte Ratio; CAR: C-reactive Protein-to-Albumin Ratio.

**Table 5:** Association between inflammatory biomarkers and risk of disease progression in AIS.

Study	Definition of Progression	Predictive Variables	Biomarkers Involved	Main Findings
[35]	Curve severity (Cobb angle; proxy)	Mechanical loading/spinal curvature	MMP-3, MMP-13, IL-1β, proteoglycans, SLRPs	Degenerative phenotype increases with severity; OA-like changes in severe AIS
[36]	Severe curve (>70° Cobb)	Cobb angle subgroup analysis	ESR1 T-DMR2 methylation	Higher methylation associated with greater severity; suggests role in progression
[37]	Progression to severe scoliosis (Cobb ≥ 45° at maturity)	Machine-learning (RFM)	miR-1-3p, miR-19a/b-3p, miR-133b, miR-143-3p, miR-148b-3p	100% accuracy, sensitivity, and specificity in predicting severe AIS
[38]	Curve severity (proxy)	Cobb angle	Leptin, OC, NTx	Significant association with severity
[39]	Severe curve requiring surgery (>40°)	Gene expression profiling	Wnt pathway genes, PCDH10, FBN genes	Tissue-specific patterns; no validated predictive biomarker
[40]	Bone metabolism (indirect)	Bone turnover, BMD	TRAP5b	Increased bone turnover may contribute to progression risk
[41]	Bone metabolism impairment (indirect)	BMD	miR-130b-3p	Increased expression linked to impaired osteogenesis
[42]	Curve severity (proxy)	Cobb angle	OCN	Lower OCN associated with greater severity
[43]	Curve severity/AIS diagnosis (proxy)	Multivariate logistic regression (clinical + molecular variables)	miR-96-5p	Improves prediction model (AUC up to 0.752); associated with AIS presence and severity
[44]	Not assessed longitudinally	Metabolic response (insulin/glucose), myogenesis	DPP-4	No predictive model; metabolic dysfunction may contribute to AIS development
[45]	Severity used as proxy	miRNA expression signature	miR-122-5p, miR-27a-5p, miR-223-5p, miR-1306-3p	High diagnostic accuracy (AUC = 0.95)
[46]	Severity used as proxy	Epigenetic regulation of chondrocytes	SUV39H1, H3K9me3, miR-15a, Bcl-2	Epigenetic activation promotes proliferation; may contribute to progression
[47]	AIS presence (no progression assessment)	Case–control comparison	Circulating cell-free DNA (Ccf-nDNA ↓; ccf-mtDNA variable)	Altered circulating DNA observed; limited predictive value
[48]	Curve severity (proxy)	Cobb angle	DPP-4 activity	Lower DPP-4 activity associated with severe curves (>50°)
[49]	Curve severity (proxy)	Cobb angle	FAP, CD23	Lower protein levels associated with greater severity
[50]	AIS vs. controls (no progression assessment)	Body composition, leptin signaling	Leptin ↓, FLI ↓, sOB-R ↑	Altered leptin bioavailability; no direct progression prediction
[51]	Curve severity (proxy)	Cobb angle	miR-941, miR-151a-3p, miR-148b-5p	Higher miRNA levels associated with severe AIS
[52]	Curve severity (proxy)	Hormonal + inflammatory markers	Androgen axis, AR, IL-6, MMP-13, STAT3	Hormonal imbalance and inflammation linked to degeneration and severity
[53]	Osteopenia (proxy for progression risk)	Ghrelin, BMD, BMI	Ghrelin, RANKL/OPG, RUNX2	Bone fragility and altered signaling linked to progression risk
[54]	Bone mass/severity (proxy)	BMD, Cobb angle	Adiponectin	Higher adiponectin associated with low bone mass and severity
[55]	Risk stratification (final Cobb angle): low ≤ 25°, medium 25–35°, high ≥ 35°	Logistic regression (miRNA signature)	7-miRNA panel; miR-4451	Predictive model (AUC = 0.83); miR-4451 reduced in high-risk group
[56]	Postoperative inflammatory response	Surgical status	IL-6, IL-1β, TNF-α	Increased inflammatory markers post-surgery; not related to progression
[57]	Curve severity (proxy)	Cobb angle	Gherelin	Higher ghrelin associated with greater severity
[58]	Severity (proxy)	Molecular expression	miR-145, CTNNB1, SOST, OPG	Altered Wnt signaling and osteocyte dysfunction linked to severity
[59]	Progression (>6° increase or Cobb ≥ 40°)	Serum levels + experimental model	Spermidine, LBX1, ODC1, SAT1	Low spermidine predicts progression; LBX1 downregulation worsens curves
[60]	Curve severity	Cobb angle	CRP, neutrophils, lymphocytes, monocytes, platelet count, CAR	Inflammatory markers significantly associated with severity
[61]	OA progression/severity (proxy)	TLR activation, cytokine expression	M-CSF, RANKL, GM-CSF, IL-1, IL-6	Increased osteoclastogenesis and inflammation linked to severity
[62]	Curve severity (proxy)	Genetic + expression analysis	SOCS3, rs4969198	Reduced SOCS3 and GG genotype associated with more severe curves
[63]	Curve severity (proxy)	miRNA + EV profiling	miR-30 family, EV proteins (SAA1, CFL1)	Severe AIS associated with distinct circulating signature; EVs impair osteogenesis

Abbreviations = AIS: Adolescent Idiopathic Scoliosis; OC: Osteocalcin; NTx: N-terminal telopeptide; Wnt: Wnt signaling pathway; PCDH10: Protocadherin 10; FBN: Fibrillin genes; ESR1: Estrogen Receptor 1; LBX1: Ladybird Homeobox 1; ODC1: Ornithine Decarboxylase 1; SAT1: Spermidine/Spermine N1-acetyltransferase 1; miRNA (miR): microRNA; AUC: Area Under the Curve; IL-1/IL-6: Interleukin-1/Interleukin-6; MMP: Matrix Metalloproteinase; SLRP: Small Leucine-Rich Proteoglycans; BMD: Bone Mineral Density; DPP-4: Dipeptidyl Peptidase-4; SOCS3: Suppressor of Cytokine Signaling 3; TLR: Toll-Like Receptor; M-CSF: Macrophage Colony-Stimulating Factor; GM-CSF: Granulocyte-Macrophage Colony-Stimulating Factor; RANKL: Receptor Activator of Nuclear Factor κB Ligand; OPG: Osteoprotegerin; AR: Androgen Receptor; STAT3: Signal Transducer and Activator of Transcription 3; RUNX2: Runt-related transcription factor 2; CTNNB1: β-catenin gene; SOST: Sclerostin; EVs: Extracellular Vesicles; CRP: C-reactive protein; CAR: C-reactive protein-to-albumin ratio; ↓: decrease; ↑: increase.

**Table 6:** Correlations between inflammatory biomarkers and clinical, anthropometric, and metabolic parameters in AIS.

Reference	Biomarker 1	Biomarker 2	Type of Correlation	Significance
[35]	Cell density	Facet loading asymmetry (concave vs. convex)	Positive (higher cellularity in AIS facets)	*p* < 0.0001
	Ki-67	Cell density	Positive	
	MMP-13	AIS tissue status	Increase in AIS	*p* < 0.001
	Decorin/Chondroadherin fragmentation	Cobb angle	Threshold effect (>70°)	Severity-dependent
	IL-6	AIS status	No correlation	Not significant
[36]		ESR1 expression (concave muscle)		
	ESR1 T-DMR2 methylation	Cobb angle	Positive	*p* < 0.05
	ESR1 T-DMR1 methylation		No correlation	Not significant
	ESR1 expression			
[37]	miR-18a-3p	Severe AIS phenotype (female)	Positive	*p* < 0.05
	miR-103a-3p	Male non-progressors vs. controls	Negative	
	miR-551b-3p	Moderate progression (male)	Positive	
	6-miRNA panel	Cobb angle severity	Strong positive (model-based)	AUC = 1.00
[38]	Leptin	NTx	Negative	*p* < 0.05
		BMI z-score	Positive	*p* < 0.001
		FAT mass (%)		*p* < 0.000001
[39]	Gene expression profiles	Cobb angle	Mixed (positive and negative)	*p* ≤ 0.05 (inconsistent)
		Risser stage		
	miRNAs	Clinical parameters	No correlation	Not significant
[40]	BAP, TRAP5b	BFR/BS	Positive	*p* = 0.002
	TRAP5b	High bone turnover		*p* = 0.032
[41]	miR-130b-3p	Bone mass	Negative	*p* < 0.05
[42]	Osteocalcin	Cobb angle	Negative	Significant
[43]	miR-96-5p	Body weight	Negative	*p* < 0.05
		Femoral neck aBMD		*p* < 0.01
		Bone microarchitecture (vBMD, BV/TV)		Significant
		Cobb angle	Positive	
[44]	DPP-4	BMI	Positive	*p* = 0.01
		Cobb angle	No correlation	*p* = 0.27
		BMI (replication cohort)		*p* = 0.89
		STAT1	Positive	*p* = 0.005
[45]	miRNA signature	Clinical variables	Not systematically evaluated	Not reported
	Pathway targets (Wnt/BMP/SMAD)	Bone metabolism pathways	Functional association	*p* < 0.001
[46]	H3K9me3	miR-15a	Negative	*p* < 0.05
	SUV39H1	H3K9me3	Positive	
	miR-15a	Bcl2	Negative	
	Bcl2	Chondrocyte proliferation (PCNA/EdU)	Positive	
	miR-15a	Chondrocyte proliferation	Negative	
[47]	ccf mtDNA	Sex	Higher in females	*p* = 0.012
		Lenke type	Higher in Lenke type 5 vs. controls	*p* = 0.024
	ccf nDNA	Lenke type	Lower in Lenke type 1 vs. controls	*p* = 0.046
[48]	DPP-4 activity	Cobb angle	Negative	Significant (*p* < 0.05 for severe curves)
		BMI	No correlation	Not significant
[49]	B2M	Age	Negative	*p* < 0.05
		FAP	Positive	*p* < 0.01
	CD23	Age	Negative	
		B2M, FAP	Positive	
[50]	sOB-R	BFM	Negative	*p* = 0.004
		% BF		*p* = 0.027
		FFM		*p* < 0.001
		SMM		
	FLI	BFM	Positive	*p* < 0.001
		% BF		
		FFM		*p* = 0.001
		SMM		
		% SMM	Negative	*p* < 0.001
[51]	miR-151a-3p	Disease severity	Positive	Significant
		GREM1 expression	Negative	
[52]	Androgen	IL-6	Negative	*p* < 0.05
		AR	Positive	*p* < 0.05
	IL-6	MMP-13		
		STAT3 phosphorylation		
		Chondrocyte proliferation	Negative	
	rs6259 SNP	Serum androgen levels	Genetic association	
[53]	Ghrelin	BMI	Negative	*p* < 0.05
		BMD		
		Osteopenia	Positive	*p* < 0.01
	RANKL/OPG ratio	Osteoclast activity		
	RUNX2	AIS status	Negative	*p* < 0.05
[54]	Adiponectin	BMD	Negative	*p* < 0.01
[55]	miRNA signature score	Cobb angle/risk group	Positive (model-based)	*p* < 0.05
	miR-4451	Risk severity	Negative	*p* < 0.01
	miRNA score	BMI/sex/menarche	No correlation	Not significant
[56]	IL-6	Surgical stress	Positive	Significant
	CRP	Inflammatory response		
[57]	Ghrelin	Age	Negative	*p* < 0.05
		cBMI, menstrual status		*p* < 0.001
		Risser stage		*p* < 0.01
	Leptin	Age, cBMI	Positive	*p* < 0.05
		Height		*p* < 0.001
		Weight, Risser stage		*p* < 0.01
[58]	miR-145	CTNNB1 (β-catenin)	Positive	*p* < 0.05
		SOST	Negative	*p* = 0.038
		OPG		*p* = 0.034
		OPN		*p* = 0.019
	CTNNB1	Osteocyte markers	Positive	*p* < 0.05
[59]	LBX1	Cobb angle	Negative	*p* < 0.05
		Myogenic markers (PAX7, MYOD, MYOG)	Positive	*p* < 0.05
	Spermidine	Curve progression	Negative	*p* < 0.05
	ODC1/SAT1	LBX1 expression	Positive	*p* < 0.05
[60]	PLR	Cobb angle	Weak/inconsistent	Partially significant
[61]	M-CSF	OA grade	Positive	*p* < 0.0001
		Intervertebral rotation		
		Sagittal/coronal angles	No correlation	Not significant
	TLR2 expression	Cytokine production	Positive	*p* < 0.05
[62]	SOCS3 expression	Cobb angle	Negative	r = 0.472, *p* = 0.014
	SOCS3 expression (concave vs. convex)	Curve severity		*p* < 0.05
	rs4969198 genotype	SOCS3 expression		
[63]	miR-30 family	Osteogenic markers (RUNX2, ALPL)	Negative	*p* < 0.05
	EV miR-30	hMSC mineralization		
	EV miRNA content	SAA1/CFL1	Positive (AIS-specific)	
	Severe AIS phenotype	miR-30 expression	Positive	
	BMI	miRNA signature	No correlation	Not significant

Abbreviations = AIS: Adolescent Idiopathic Scoliosis; BMI: Body Mass Index; BMD: Bone mineral density; aBMD/vBMD: Areal/Volumetric Bone Mineral Density; BV/TV: Bone Volume/Total Volume; NTx: N-terminal telopeptide; miRNA (miR): microRNA; ESR1: Estrogen Receptor 1; STAT1/STAT3: Signal Transducer and Activator of Transcription 1/3; LBX1: Ladybird Homeobox 1; ODC1: Ornithine Decarboxylase 1; SAT1: Spermidine/Spermine N1-acetyltransferase 1; MMP: Matrix Metalloproteinase; IL-6: Interleukin-6; Bcl2: B-cell lymphoma 2; SOCS3: Suppressor of Cytokine Signaling 3; OA: Osteoarthritis; M-CSF: Macrophage Colony-Stimulating Factor; TLR2: Toll-Like Receptor 2; RANKL: Receptor Activator of Nuclear Factor κB Ligand; OPG: Osteoprotegerin; RUNX2: Runt-related transcription factor 2; CTNNB1: β-catenin gene; SOST: Sclerostin; OPN: Osteopontin; ALPL: Alkaline Phosphatase; DPP-4: Dipeptidyl Peptidase-4; SAA1: Serum Amyloid A1.

**Table 7:** Summary of evidence strength for biomarker categories associated with AIS severity and progression.

Biomarker Category	Main Outcomes Investigated	Consistency of Findings	Level of Evidence *
Inflammatory cytokines (IL-6, IL-17, TNF-α, etc.)	Severity	Multiple studies reporting significant associations with curve severity	Moderate
miRNAs and epigenetic markers	Severity and progression	Multiple studies identified associations, but external validation remains limited	Moderate
Bone metabolism markers (osteocalcin, TRAP5b, BAP, RANKL/OPG)	Severity	Reproducible associations across several independent studies	Moderate
Metabolic and endocrine markers (leptin, ghrelin, adiponectin, DPP-4)	Severity	Findings generally support an association, although results remain heterogeneous	Limited–Moderate
Extracellular vesicle-associated biomarkers	Severity	Preliminary evidence from a small number of studies	Limited
mtDNA biomarkers	Severity	Evidence derived from a single exploratory study	Preliminary
Spermidine	Progression	Evidence derived from a single longitudinal study	Preliminary
Osteocyte network and tissue-related biomarkers	Severity	Exploratory findings requiring independent validation	Preliminary

* Level of evidence reflects the consistency and replication of findings across the included studies and should not be interpreted as a formal GRADE assessment.

## Data Availability

The original contributions presented in this study are included in the article/Supplementary Materials. Further inquiries can be directed to the corresponding author(s).

## Supplementary Materials

The following supporting information can be downloaded at: https://www.mdpi.com/article/10.3390/cells15121044/s1, Table S1: Combination of free-vocabulary and/or Medical Subject Headings (MeSH) terms for the identification of studies.

## Abbreviations

The following abbreviations are used in this manuscript:

AIS	Adolescent idiopathic scoliosis
PICOS	Population, Intervention, Comparison, Outcomes, Study design
PRISMA	Preferred Reporting Items for Systematic Reviews and Meta-Analyses
MeSH	Medical Subject Headings
ROBINS-I	Risk Of Bias In Non-randomized Studies of Interventions
IL	interleukin
MMP	matrix metalloproteinase
NLR	neutrophil-to-lymphocyte ratio
CAR	C-reactive protein/albumin ratio
TNF-α	Tumor Necrosis Factor alpha
DPP-4	Dipeptidyl peptidase-4
TLRs	Toll-like receptors
miRNAs	microRNAs
ESR1	estrogen receptor 1
SUV39H1	Histone-lysine N-methyltransferase
H3K9me3	Histone H3 Lysine 9 trimethylation
SOCS3	Suppressor of Cytokine Signaling 3
RUNX2	Runt-related transcription factor 2
RANKL	Receptor Activator of Nuclear Factor kappa-B Ligand
OPG	Osteoprotegerin
TRAP5b	Tartrate-resistant acid phosphatase 5b
STAT3	Signal Transducer and Activator of Transcription 3
