# Supplementary material for: Circulating and Tissue Biomarkers Associated with Disease Severity and Progression in Adolescent Idiopathic Scoliosis: A Systematic Review
Source: Cells. 2026 Jun 6;15(12):1044. doi: 10.3390/cells15121044 (PMC13297147; doi:10.3390/cells15121044)
Supplement: Supplementary file 1 [file cells-15-01044-s001.zip › cells-4357305-supplementary.pdf]

Table S1. Combination of free-vocabulary and/or Medical Subject Headings (MeSH) terms for the identification of studies.

Pubmed: ("Scoliosis"[MeSH] OR "adolescent idiopathic scoliosis" OR AIS OR "idiopathic scoliosis") AND ("Biomarkers"[MeSH] OR "Inflammation Mediators"[MeSH] OR "Cytokines"[MeSH] OR cytokines OR interleukin* OR "inflammatory biomarkers" OR "immune markers" OR microRNA OR miRNA OR "non-coding RNA" OR "epigenetic markers") AND ("Disease Progression"[MeSH] OR "Severity of Illness Index"[MeSH] OR "Cobb angle" OR severity OR "curve severity" OR progression OR "curve progression" OR "progression risk" OR surgery OR "surgical indication" OR "Risser sign" OR "skeletal maturity") AND ("Adolescent"[MeSH] OR adolescent* OR child*) NOT (animal* OR rat OR mouse)
Scopus: ("adolescent idiopathic scoliosis" OR AIS OR "idiopathic scoliosis") AND ("inflammatory biomarkers" OR biomarkers OR cytokines OR interleukin* OR microRNA OR miRNA OR "non-coding RNA" OR "epigenetic markers") AND ("Cobb angle" OR severity OR "curve severity" OR progression OR "curve progression" OR "progression risk" OR surgery OR "Risser sign" OR "skeletal maturity")
Web of Science Core Collection: ("adolescent idiopathic scoliosis" OR AIS OR "idiopathic scoliosis") AND ("inflammatory biomarkers" OR biomarkers OR cytokines OR interleukin* OR microRNA OR miRNA OR "non-coding RNA" OR "epigenetic markers") AND ("Cobb angle" OR severity OR "curve severity" OR progression OR "curve progression" OR "progression risk" OR surgery OR "Risser sign" OR "skeletal maturity")
